# Placental vascular alterations are associated with early neurodevelopmental and pulmonary impairment in the rabbit fetal growth restriction model

**DOI:** 10.1038/s41598-022-22895-6

**Published:** 2022-11-16

**Authors:** Ignacio Valenzuela, David Basurto, Yannick Regin, Andre Gie, Lennart van der Veeken, Simen Vergote, Emma Muñoz-Moreno, Bartosz Leszczynski, Birger Tielemans, Greetje Vande Velde, Jan Deprest, Johannes van der Merwe

**Affiliations:** 1 Department of Development and Regeneration, Cluster Woman and Child, Group Biomedical Sciences, KU Leuven, Herestraat 49, Box 805, 3000 Leuven, Belgium; 2 Department of Paediatrics and Child Health, Faculty of Medicine and Health Sciences, Stellenbosch University and Tygerberg Hospital, Cape Town, South Africa; 3 Magnetic Resonance Imaging Core Facility, Institut d’Investigacions Biomèdiques August Pi i Sunyer (IDIBAPS), Barcelona, Spain; 4 Department of Medical Physics, Marian Smoluchowski Institute of Physics, Faculty of Physics, Astronomy and Applied Computer Science, Jagiellonian University, Łojasiewicza 11, 30-348 Kraków, Poland; 5 Biomedical MRI/MoSAIC, Faculty of Medicine, Department of Imaging and Pathology, KU Leuven Herestraat 49, 3000 Leuven, Belgium; 6 Department of Obstetrics and Gynecology, Division Woman and Child, University Hospitals Leuven, Herestraat 49, 3000 Leuven, Belgium

**Keywords:** Intrauterine growth, Preclinical research, Experimental models of disease, Paediatric research

## Abstract

Fetal growth restriction is one of the leading causes of perinatal mortality and morbidity and has consequences that extend well beyond the neonatal period. Current management relies on timely delivery rather than improving placental function. Several prenatal strategies have failed to show benefit in clinical trials after promising results in animal models. Most of these animal models have important developmental and structural differences compared to the human and/or are insufficiently characterized. We aimed to describe placental function and structure in an FGR rabbit model, and to characterize the early brain and lung developmental morbidity using a multimodal approach. FGR was induced in time-mated rabbits at gestational day 25 by partial uteroplacental vessel ligation in one horn. Umbilical artery Doppler was measured before caesarean delivery at gestational day 30, and placentas were harvested for computed microtomography and histology. Neonates underwent neurobehavioral or pulmonary functional assessment the day after delivery, followed by brain or lung harvesting, respectively. Neuropathological assessment included multiregional quantification of neuron density, apoptosis, astrogliosis, cellular proliferation, and oligodendrocyte progenitors. Brain region volumes and diffusion metrics were obtained from ex-vivo brain magnetic resonance imaging. Lung assessment included biomechanical tests and pulmonary histology. Fetal growth restriction was associated with labyrinth alterations in the placenta, driven by fetal capillary reduction, and overall reduced vessels volume. FGR caused altered neurobehavior paralleled by regional neuropathological deficits and reduced fractional anisotropy in the cortex, white matter, and hippocampus. In addition, FGR kittens presented functional alterations in the peripheral lung and structurally underdeveloped alveoli. In conclusion, in a uteroplacental insufficiency FGR rabbit model, placental vascular alterations coincide with neurodevelopmental and pulmonary disruption.

## Introduction

Fetal Growth Restriction (FGR) refers to the inability of a fetus to achieve its genetic growth potential^1^. It is one of the main contributors to perinatal mortality and morbidity, and it is associated with almost 50% of stillbirth cases worldwide^2–4^. Beyond the neonatal period, it causes cognitive and behavioral impediment in childhood^5^ and metabolic, pulmonary, and cardiovascular disease in adult life^6,7^. The main cause of FGR is uteroplacental insufficiency (UPI), a condition whereby a defective placental development leads to an insufficient supply of oxygen and nutrients to support the developing fetus, resulting in acidosis, hypoxemia, and in more severe cases ultimately intrauterine fetal demise. There is currently no effective prenatal treatment for FGR: clinical trials have failed to deliver a safe and effective therapy, even after successful preclinical studies^8–10^. Well characterized animal models with developmental, functional, and structural similarities to the human are fundamental to understand the consequences of FGR and to design new therapeutic strategies aimed at ameliorating them.

Several animal models have been described but so far no single animal model mimics the entire spectrum of human FGR^11^. In recent years, the rabbit has been established as a well-rounded, versatile model for perinatal conditions^12–18^. Like humans and rodents, they have discoid, haemochorial placentas, in which maternal blood is in direct contact with the trophoblast. Furthermore, only two trophoblast layers separate maternal and fetal blood spaces (hemo-dichorial placenta), closing the gap between the murine hemo-trichorial and the human hemo-monochorial placentas^19^. Trophoblast volumes, placental glucose transporters, and other pregnancy-specific changes in the rabbit have also been shown to mimic the clinical scenario^20^.

In addition to the morphological and functional placental similarities, rabbits resemble humans closer than other species in terms of neurodevelopment. Neurogenesis starts in the first trimester, and white matter maturation begins around birth to continue through the first year of life^21,22^. Pulmonary development is also a perinatal process, with alveolarization beginning prior to birth, so that at term rabbit lungs are in the terminal air sac stage^23–25^. In addition to that, rabbits have several practical benefits; they are non-seasonal in mating habits, have a short gestation period with a large litter size, and are suitable for foster care and follow-up assessment.

Our group has utilized and refined a robust neurodevelopmental and pulmonary multimodal evaluation platform to assess newborn and preadolescent rabbits and phenotype the effects of bronchopulmonary dysplasia^26–29^, prematurity^30,31^, and congenital diaphragmatic hernia^32,33^. Herein, we characterize the consequences of FGR induced by uteroplacental vessel ligation (UPVL) in placental microvasculature, and early brain and lung development in the rabbit model.

## Results

### UPVL alters labyrinth zone vascularization, increases mortality, and induces FGR

Twenty-two does delivered 65 live kittens from the ligated horns, and 86 from the control horns. UPVL resulted in higher intrauterine mortality (p < 0.0001) and a significant reduction in body weight (p < 0.0001), placental weight (p < 0.0001), and fetal/placental weight ratio (p < 0.0001; Table 1). Umbilical artery doppler parameters (PSV, EDV, VTI, MV, PR and RI) were not significantly different between groups (Supplementary Table S1).

**Table 1:** Survival and biometrics.

n = 22 does	FGR	Control	P-value
Survival	65/130 (50%)	86/97 (89%)	< 0.0001
Birth weight (g)	32.93 ± 0.93	44.29 ± 1.05	< 0.0001
Placental weight (g)	5.33 ± 0.15	6.15 ± 0.15	< 0.0001
Brain weight (g)	1.46 ± 0.04	1.74 ± 0.05	< 0.0001
Brain/body weight ratio	0.031 ± 0.002	0.027 ± 0.004	0.0036
Fetal/placental weight ratio	6.41 ± 1.07	7.30 ± 1.07	< 0.0001

Survival and birth weight were assessed 4 h after caesarian delivery. Brain weight was measured at PND1. Data were analysed with Fischer’s exact test or using a linear mixed-effects model. Values are displayed as mean ± SE. FGR: fetal growth restriction.

**Figure 1:**
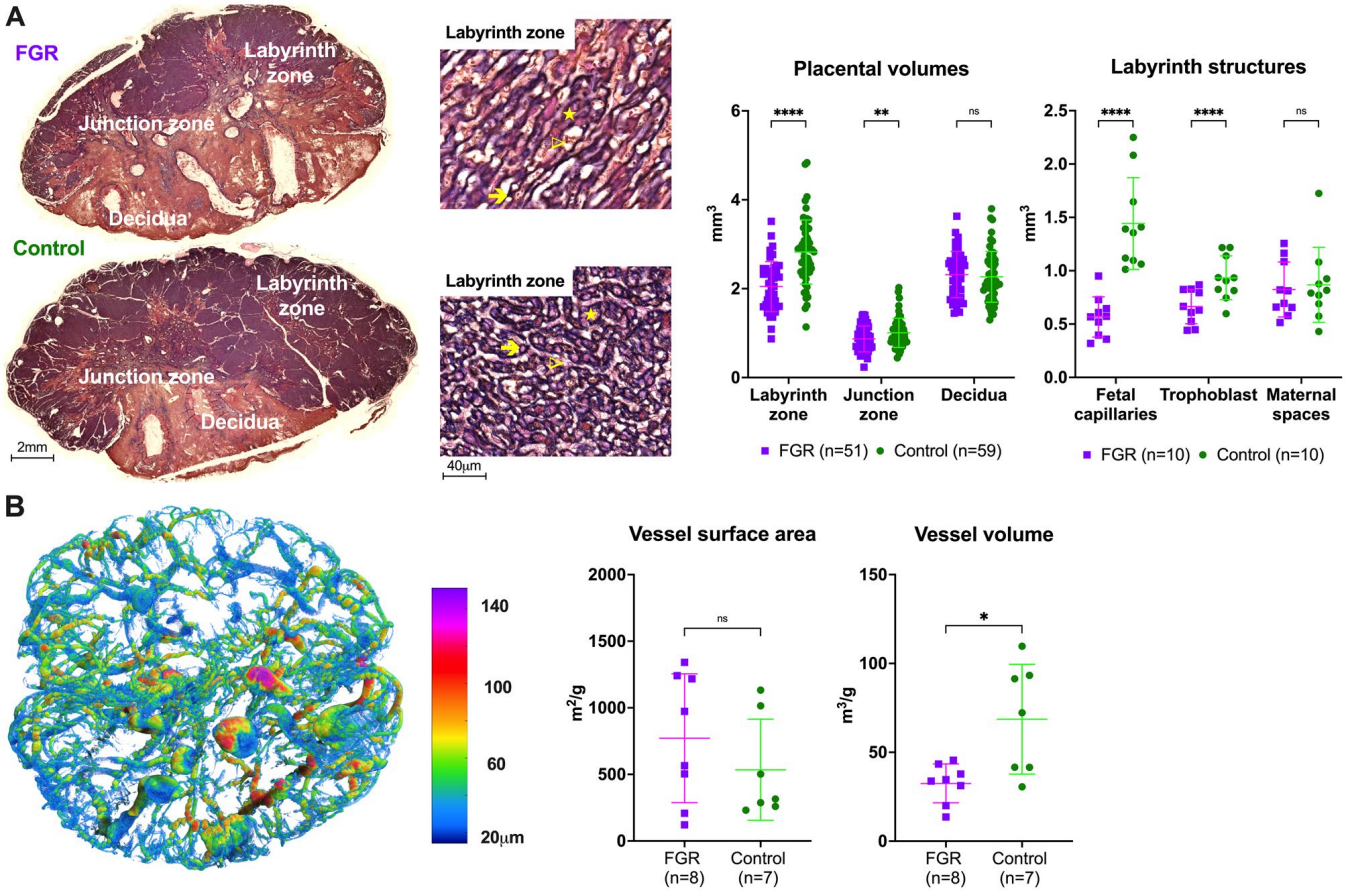
Placental histology and microcomputed tomography. (**A**) Placentas with cytokeratin/lectin double staining, divided by placental zones (left images). Within the labyrinth (zoomed, right), fetal capillaries (arrow), maternal blood spaces (arrowhead) and trophoblast (star) were grid counted to calculate relative and absolute volume densities. For placental volumes, 51 FGR and 59 control placentas were used from 20 litters. For labyrinth structures, 10 FGR and 10 control placentas were used from 10 litters (1 placenta per horn, per litter). (**B**) Representative image of a placental microcomputed tomography after color coding according to vessel diameter. Data were analysed using a linear mixed-effects model and displayed as mean ± SD with significance as *0.05 ≥ p > 0.01; **0.01 ≥ p > 0.001.; ***0.001 > p > 0.0001; ****p < 0.0001.

At histological assessment placentas from FGR kittens had a significantly smaller labyrinth (p < 0.0001) and junction zone (p = 0.003) when compared to controls (Fig. 1A). Within the labyrinth zone, the proportion of fetal capillaries (FC) was significantly reduced (p < 0.0001) in FGR placentas, and the proportion of maternal blood spaces (MBS) significantly increased (p < 0.0001) (Supplementary Table S2). FC (p < 0.0001) and trophoblast volumes (p = 0.0001) were significantly decreased in FGR placentas, while MBS volumes were not significantly different when compared to controls (Fig. 1A). The area of fibrosis in the decidua was not significantly different between groups (Supplementary materials, Fig. S1). Microvasculature assessment by computerized tomography showed FGR placentas had significantly reduced vessel volume after correction for placental weight (p = 0.015) (Fig. 1B). The vessel surface area and the distribution of vessels according to their diameter was not significantly different between groups.

### FGR is associated with early neurodevelopmental impairment

**Figure 2:**
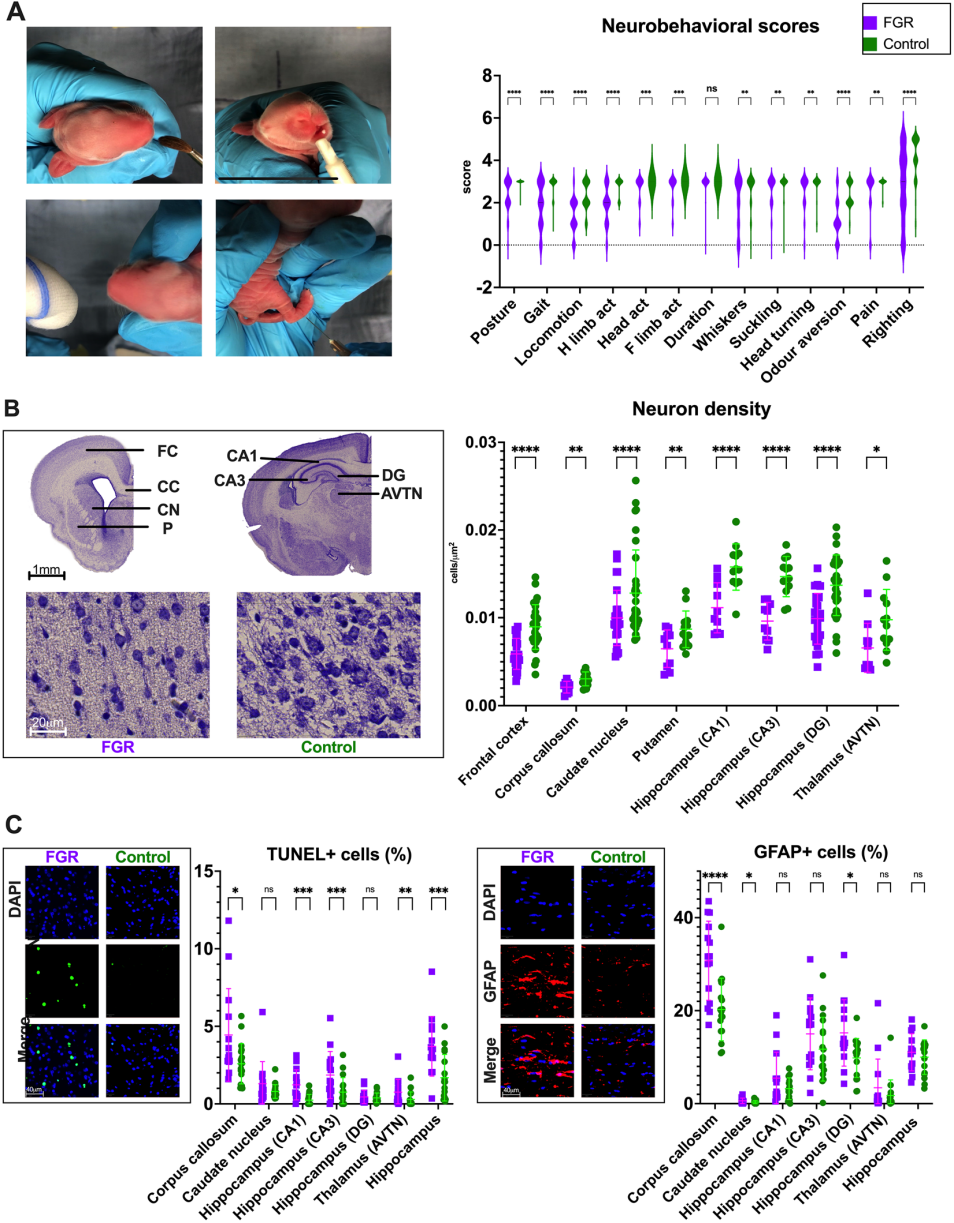
Brain assessment in postnatal day 1 rabbits. (**A**) Neurobehavioral tests (left) and grouped scores (right) from 43 FGR and 61 control subjects from 16 litters. (**B**) Neuron density was assessed in cresyl violet stained slides in the depicted areas (top images). Bottom images show neuron density in the frontal cortex of FGR and control brains. To the right, neuron density from 27 FGR and 29 control subjects from 16 litters. (**C**) Representative images of apoptosis (TUNEL), and astrogliosis (GFAP) in hippocampus and corpus callosum, respectively. Data from 14 FGR and 19 control subjects from 11 litters. *CA1* cornu ammonis 1, *CA3* cornu ammonis 3, *DG* dentate gyrus, *AVTN* anteroventral thalamic nuclei. Data were analysed using a linear mixed-effects model and displayed as mean ± SD with significance as *0.05 ≥ p > 0.01; **0.01 ≥ p > 0.001.; ***0.001 > p > 0.0001; ****p < 0.0001.

FGR neonates had a significant reduction in brain weight (p < 0.0001) and increased brain to body weight ratio (p = 0.0036) at birth (Table 1). FGR was also associated with motor and sensory deficits on neurobehavioral assessment (NBA), as displayed in Fig. 2A. FGR kittens had abnormal posture, gait, and locomotion, but activity duration was similar to controls. Additionally, FGR led to hampered cranial nerves activity, pain response, and righting reflex.

### FGR kittens have lower multiregional neuron density, associated with increased apoptosis and astrogliosis, and reduced oligodendrocyte precursor cells

As shown in Fig. 2B, FGR led to a significant reduction in neuron density in the frontal cortex (FCx, p < 0.0001), corpus callosum (CC, p = 0.0059), caudate nucleus (CN, p < 0.0001), putamen (p = 0.0018), cornu ammonis 1 (CA1; p < 0.0001), cornu ammonis 3 (CA3; p < 0.0001), dentate gyrus (DG, p < 0.0001), and anteroventral thalamic nuclei (AVTN, p = 0.0170). This coincided with an increased proportion of TUNEL-positive cells in the CC (p = 0.0127), CA1 (p = 0.0005), CA3 (p = 0.0004), AVTN (p = 0.002) and whole hippocampus (p = 0.0003) in FGR brains (Fig. 2C). Additionally, rabbits from the FGR group had a higher proportion of GFAP-positive cells in the CC (p < 0.0001), CN (p = 0.0305) and DG (p = 0.0168) (Fig. 2C). NG-2-positive cells were significantly decreased in the FCx (p = 0.0093), CC (p = 0.0423) and CN (p = 0.0245) (Supplementary materials, Fig. S2, Table S3). No significant differences were found in Ki67-positive cells in the same regions (Fig. S3, Table S3).

### Regional neuropathological changes coincide with MRI volumetric and diffusion weighted imaging

T1-derived volumetric data from magnetic resonance imaging (MRI) showed that FGR rabbits had an overall lower absolute brain volume. However, after normalizing the regional volumes to their respective total brain volume, no differences were present between the two groups in the volume of specific brain regions.

**Figure 3:**
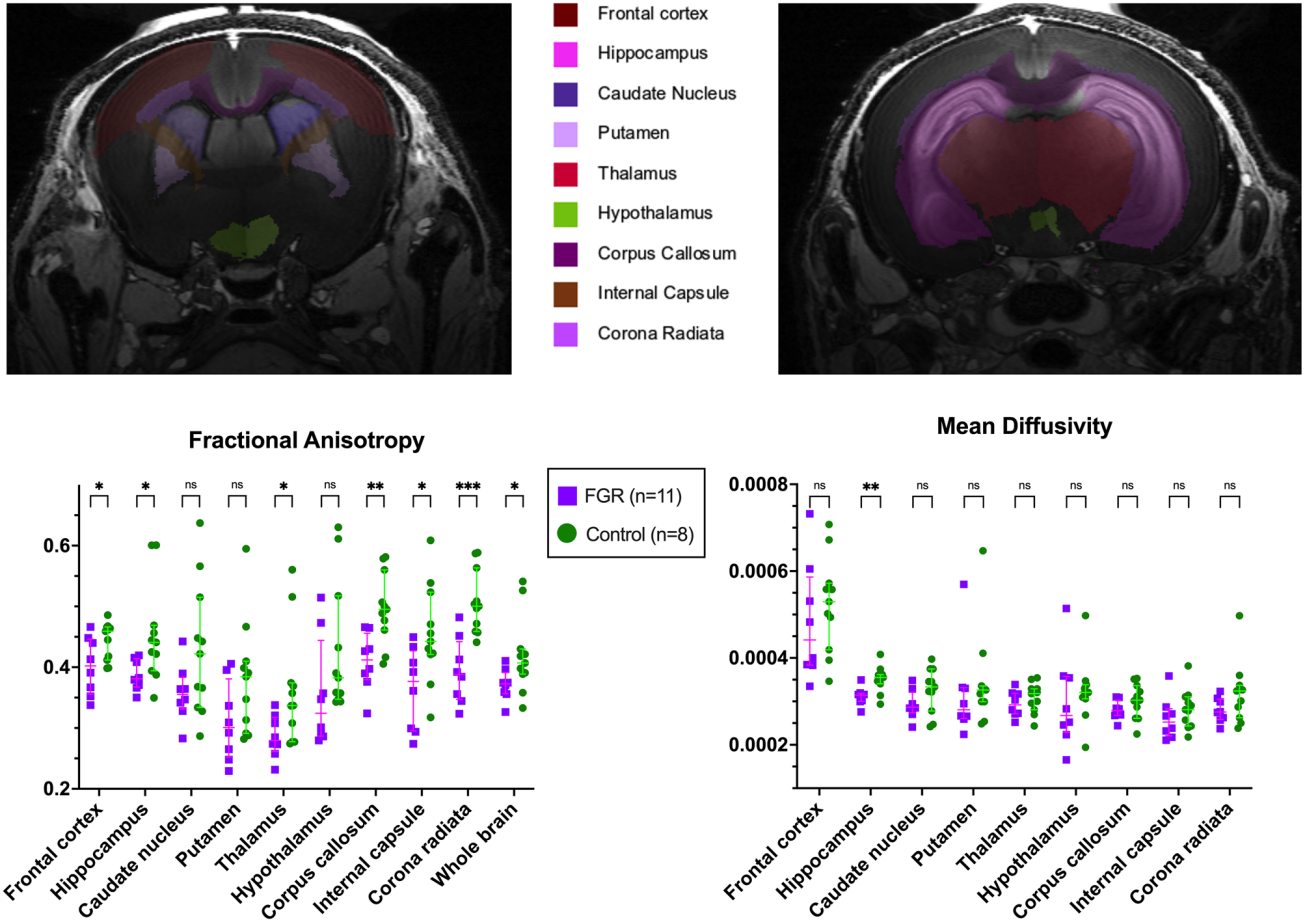
Ex vivo brain diffusion tensor metrics at postnatal day 1 from 11 FGR and 8 control brains, from 6 litters. Data were analysed using a linear mixed-effects model and displayed as mean ± SD with significance as *0.05 ≥ p > 0.01; **0.01 ≥ p > 0.001.; ***0.001 > p > 0.0001; ****p < 0.0001.

Differences on diffusion tensor metrics also paralleled histological findings. Fractional anisotropy (FA) was globally reduced (p = 0.0444), and additionally significantly lower in the FCx (p = 0.0374), CC (p = 0.0032), internal capsule (IC, p = 0.0202), corona radiata (CR, p = 0.0003), hippocampus (p = 0.0259), thalamus (p = 0.0326) in FGR rabbits, while there was no significant difference in the CN, putamen, and hypothalamus (Fig. 3, Table S4). Mean diffusivity (MD) was only found significantly reduced in the hippocampus (p = 0.004) (Fig. 3, Table S4).

### FGR leads to significant alteration of peripheral lung mechanics and compliance

**Figure 4:**
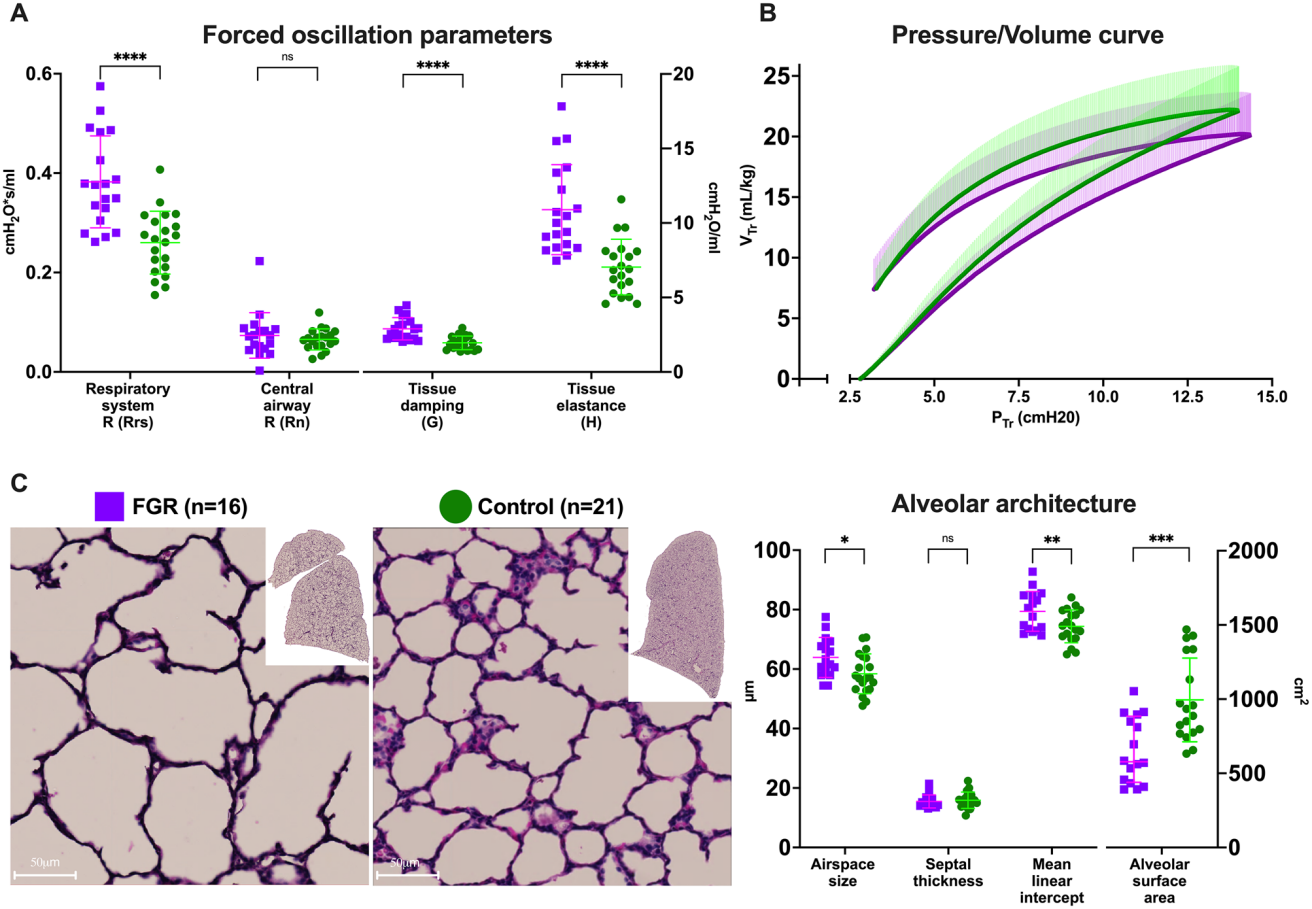
Pulmonary assessment in postnatal day 1. (**A**, **B**) Pulmonary function tests results from 19 FGR and 21 control subjects from 6 litters. (**C**) Representative images of histological H&E-stained slides (left) and alveolar architecture data (right) from 16 FGR and 21 control subjects from 6 litters. Data were analysed using a linear mixed-effects model and displayed as mean ± SD with significance as *0.05 ≥ p > 0.01; **0.01 ≥ p > 0.001.; ***0.001 > p > 0.0001; ****p < 0.0001.

Lung function was significantly reduced in FGR animals. Forced oscillation tests showed increased tissue damping (p < 0.0001), tissue elastance (p < 0.0001), respiratory system resistance (p < 0.0001) and decreased dynamic compliance (p < 0.0001) in FGR lungs, while central airway resistance was not significantly different between groups (Fig. 4A, Table S5). In pressure–volume perturbations, FGR lungs had significantly reduced hysteresis (p = 0.0004) and static compliance (p = 0.0018) compared to controls (Fig. 4B, Table S5).

### FGR does not influence overall lung volume, but disrupts alveolar development

Both groups had similar lung sizes when corrected by body weight, as measured by water displacement (37.21 ± 7.02 vs. 39.12 ± 5.45 mL; p = 0.1333) and inspiratory capacity (31.60 ± 5.8 vs. 34.13 ± 4.55 mL; p = 0.0823). In contrast, on histological assessment FGR lungs had significantly decreased alveolar surface area (S; p = 0.0004), increased alveolar size (Lm; p = 0.0049), and increased alveolar airspace (Lma; p = 0.0191), while septal thickness (Lmw) was similar between groups (Fig. 4C). The medial thickness of the peripheral pulmonary arteries was also comparable between groups (Supplementary materials, Fig. S4).

## Discussion

This study expands the characterization of the rabbit FGR model as previously described^12,16^ by utilizing a multimodal assessment approach, adding a description of placental microvascular changes, and subsequent early functional and structural alterations in the brain and lungs. In this model the exchange zone area -the labyrinth- and overall vessel volume in FGR placentas are significantly reduced. Upon neuropathological review, lower neuron density and higher apoptosis coincide with reduced fractional anisotropy in grey and white matter structures and are associated with neurobehavioral deficits. Furthermore, lung parenchymal functional and structural changes are demonstrated for the first time in this FGR model. Herein, the reduction in alveolar surface area accompanies the increased resistance and damping in the peripheric lungs.

Underdevelopment of the labyrinth is the most notable finding in the placenta. Decidual volume was similar between FGR and control placentas and did not present significantly higher fibrosis after UPVL, in line with previous reports using this model^34^. Labyrinth volume reduction was driven by a reduction in fetal capillaries at histological level and coincided with the reduction in fetoplacental vessel volume on microcomputed tomography. Although this is the first published experience using this technique in the rabbit, labyrinth alterations have been previously described in several other FGR models, including those induced by maternal undernutrition^35,36^, exogenous^37,38^ and endogenous^39^ glucocorticoid excess, heavy metal exposure^40^, NO synthetase inhibition^41,42^, and systemic inflammation^43^. Furthermore, labyrinth underdevelopment, and particularly fetal vessel reduction, has been associated with reduced levels of different placental vascular endothelial growth factor (*VEGF)* mRNAs^38^, and glucose transport capacity^39,44^. Future placental characterization in this model to include the expression of these and other key genes, as well a more in-depth description of the decidua and junction zone, might help unravel the complex molecular machinery behind placental development and function in uteroplacental insufficiency.

Interestingly, histological findings in the placenta were not associated with an alteration in umbilical artery doppler, in accordance with the work by Eixarch et al.^45^. Anesthesia-induced hemodynamic changes at the time of evaluation might have masked true differences between subjects. Indeed, the umbilical artery PI and RI in our study are considerably higher than those observed in normally grown, non-sedated animals at the same gestational age^46^.

The pulmonary morbidity in this study is likely the result of uteroplacental insufficiency and not directly caused by toxins or drugs, as in other models^47,48^. Moreover, the pulmonary impairment was not influenced by prematurity or postnatal lung injury, commonly present in perinatal lung assessment. The rabbit mimics human pulmonary developmental closer than other species as alveolarization is a perinatal process^49^, and not entirely prenatal as in sheep and guinea pigs, or postnatal as in rats and mice. Nonetheless, our results are consistent with findings in murine and ovine models^50–53^, and suggest FGR disrupts alveolar development and leads to biomechanical dysfunction, but does not affect lung volume. The functional impairment was observed in both forced oscillation and pressure–volume maneuvers. On the former, increased tissue damping, tissue elastance and respiratory system resistance indicate peripheral remodeling. The similar central airway resistance suggests comparable large airways caliber and is in line with the timing of the placental insult after the formation of the central airways. The decreased hysteresis and poorer compliance found on pressure–volume maneuvers indicate that FGR results in stiffer lungs and that the work of breathing is higher in this group. Structural data is in line with the biomechanical findings and coincides in a peripheral lung alteration, showing a simplified alveolar morphology, with increased airspace size and lower alveolar surface area available for gas exchange.

The molecular and cellular drivers underlying these changes are likely multiple and complex, and their description was not the purpose on this model characterization. They will, however, need to be approached and compared against findings in other models, which include dysregulation of *VEGF*^50,52^, retinoic acid receptors^54,55^, and other pathways involved in hypoxia signaling^56,57^, tissue repair, and cellular communication regulation^58^. Additionally, it will be of interest to determine if these functional and structural alterations are sustained in the longer term, or if any new pulmonary findings develop overtime.

Regarding neurodevelopmental assessment, the neurobehavioral deficit we describe is in line with previous reports in the rabbit^59,60^ and other animal models^61–63^, highlighting the cross-species nature of these findings. Furthermore, the neurobehavioral deficit correlated with lower neuron density at neuropathology and altered structural integrity at MRI. Interestingly, the latter was only significant in the frontal cortex, white matter regions, and hippocampus, yet the caudate nucleus and putamen seem to be less affected. This coincides with regional variations in brain perfusion described in the context of brain sparing in severe FGR^64^ with preferential supply of blood flow to the deep grey matter of the basal ganglia in detriment of the frontal cortex. Reduced fractional anisotropy, lower neuron density, and increased apoptosis in these regions suggest structural integrity is compromised in FGR brains even before myelination takes place^65^. Organization in neuronal population, followed by axonal and dendritic growth, increase density in these areas, thus an insult to neuronal organization might reduce FA independent of myelination. Recent work describing the significant correlation between birth weight and preoligodendrocyte density in this model^34^ suggest a disruption in the oligodendrocyte maturation process. Although the difference in immunohistochemical techniques does not allow for a direct comparison, these results are in accordance with the reduced density of NG2+-cells found in the corpus callosum in our FGR subjects. Moreover, the same group has recently described an impairment in the oligodendrocyte differentiation capacity of neural progenitor cells incubated in a novel neurosphere culture^66^.

We acknowledge our study has limitations. First, the acute induction of UPI does not mimic the slow progressive nature of FGR. However, most models with an earlier and/or more persistent induction, like maternal undernutrition or glucocorticoid excess, either fail to recreate the severity of FGR, or expose the fetus to insults not related to placental function. Similarly, attempts to reproduce the progressiveness of UPI in surgical models have failed to mimic the severity on the disease by increased fetal mortality^67,68^. Secondly, this model describes the effects of FGR in near-term neonates, even though most severe clinical cases require preterm delivery. Pulmonary and neurodevelopment are highly dependent on GA at delivery, and thus assessing near-term neonates allows us to unmask the effects of FGR from those of prematurity. Lastly, only immediate neonatal consequences of FGR are reported in the current study. We are aware that our findings need to be supplemented with observations on the mid and long-term, and that further assessment is a necessary next step into the robust characterization of the rabbit model, and we are currently working towards such characterization.

In conclusion, in the rabbit UPVL FGR model we demonstrated that placental vascular alterations lead not only to neurodevelopmental but also pulmonary deficits in the early neonatal period. A longitudinal FGR model characterization of placental, neurocognitive, and pulmonary function and structure could greatly aid in assessing the safety and efficacy of novel therapeutic strategies.

## Materials and methods

### Animal model

All animals were treated according to current guidelines for animal well-being, and experiments were approved by the Ethics Committee for Animal Experimentation of the Faculty of Medicine (P080/2019). Experiments are reported according to ARRIVE guidelines^69^. Time-mated rabbits (hybrid of Dendermonde and New Zealand White) were housed in individual cages at 21 °C, 42% humidity, with a 12-h day/night cycle and free access to food and water. Conception day was considered day 0 of pregnancy (GA = 0). At GA 25 (full term 31.5 days), does underwent induction of FGR. Briefly, rabbits were administered induction anesthesia with IM ketamine (35 mg/kg Nimatek^®^, Eurovet Animal Health BV, Bladel, The Netherlands) and xylazine (5 mg/kg XYL-M^®^ 2%, VMD, Arendonk, Belgium), and antibiotic prophylaxis (10 mg/kg Baytril^®^ 2.5% SC, Bayer, Diegem, Belgium), tocolysis (10 mg/kg Depo-Provera^®^ SC, Pfizer, Puurs, Belgium), and analgesia (0.03 mg/kg Vetergesic^®^ SC, Ceva Animal Health, Brussels, Belgium) prior to surgery. Anesthesia was maintained with a continuous IV infusion of ketamine (8–16 mg/kg/h) and xylazine (2.4–4.8 mg/kg/h), while monitoring vital signs. Following laparotomy, 30–40% of the vessels going to each placenta were ligated in one random horn with Vicryl^®^ 5-0 (Ethicon^®^, Diegem, Belgium), leaving the contralateral unligated horn as internal control. The abdomen was closed with Vicryl^®^ 2-0 and Monocryl^®^ 3-0 (Ethicon^®^, Diegem, Belgium) for fascia and skin, respectively. The surgical wound was infiltrated with levobupivacaine (2 mg/kg Chirocaine^®^, Abbvie, Wavre, Belgium) and sprayed with aluminium (Kela^®^, Hoogstraten, Belgium).

Rabbits were monitored daily until delivery by caesarian section at GA 30. Following delivery, does were euthanized using IV phenytoin/pentobarbital (140 mg/kg Euthasol^®^, Kela, Bladel, The Netherlands). Kittens were pet dried, numbered with a permanent marker, and kept in a warmed (34 °C) and humidified (55% RH) incubator. Later that day they were stimulated to urinate, weighed, and fed a commercial milk substitute (Day One, protein 30%, fat 50%; Fox Valley, Lakemoor, IL) with added probiotics (Bio-Lapis; Probiotics International, Somerset, UK) and immunoglobulins (Col-o-Cat; SanoBest, Hertogenbosch, The Netherlands). The following morning litters were allocated for brain or lung assessment.

### Placental ultrasound

At the time of caesarean delivery rabbits were anesthetized with the same protocol as for FGR induction, put on a warming pad under a warming light, and uterine horns were exposed and continuously rinsed with warm saline (37 °C). The VisualSonics VEVO 2100 (Toronto, Ontario, Canada) high-resolution micro ultrasound platform and a VisualSonics MS-250 transducer were used for image acquisition (center frequency 21 MHz; bandwidth 13–24 MHz; geometric focus 15 mm; maximum image width 23 mm; maximum image depth 30 mm; footprint 28 × 5.75 mm). Uterine artery doppler waveforms were obtained from the fetuses located closest to the ovarian end of each uterine horn. The umbilical vessels were located using color Doppler and the Doppler sample gate was placed over the umbilical artery, keeping the angle to a minimum. Peak systolic velocity (PSV), end diastolic velocity (EDV), velocity time integral (VTI), mean velocity (MV), pulsatility index (PI), and resistance index (RI) were calculated offline using the VisualSonics analysis software.

### Placental histology

After delivery, placentas (until and including the decidua) were carefully separated from the implantation sites in the uterine wall, washed in PBS, trimmed from umbilical cord and membranes, blotted dry, weighed, and immerse-fixed in 4% paraformaldehyde (PFA) for 72 h. The smallest lobe was sectioned in 2 coronal portions, embedded in paraffin blocks, and cut in 4 µm slides. 2 slides per placenta were then double stained for cytokeratin and lectin to assess trophoblast, FC, and MBS. The detailed staining protocol is added in the Supplementary materials. Another 2 slides per placenta were subjected to standard Masson’s trichrome staining to visualize collagen deposition in the decidua.

All slides were digitally scanned with the Zeiss AxioScan Z1 imaging platform (AxioScan^®^ Slide Scanner, Carl Zeiss MicroImaging GmbH, Munich, Germany).

Placental zones (labyrinth, junction, decidua) were manually delineated using QuPath software^70^ and their area relative to the total placental area was calculated. The structures within the labyrinth zone were manually quantified as described before^71^. Briefly, using a 4 × 4 point counting grid on 16 random fields at 20× magnification, the number of MBS, FC, and trophoblast cells was quantified and expressed as a percentage of the total count. Placental zone volumes and labyrinth structures volumes were calculated multiplying the relative volumes and the placental weight. To calculate the area of fibrosis in the decidua, images were manually delineated, and the selection was transferred to ImageJ (version 1.53e, Fiji, USA)^72^. Blue-stained collagen fibers were used to calculate the ratio of signal-positive area/total decidual area, expressed as percentage. A blinded observer (I.V.) performed all histological evaluations.

### Placental computed microtomography

A subset of rabbits was used for fetoplacental perfusion with a contrast agent under the same anesthesia protocol at GA30. Following laparotomy, both uterine horns were removed and immediately placed on ice. Fetuses and placentas were exposed, and a 24G cannula was inserted in the umbilical vein of each fetus, while an umbilical artery was cut open to serve as vent. Initially, warm heparinized saline was perfused, followed by a barium-based contrast solution consisting of 0.9% sodium chloride solution, barium sulfate (E-Z PAQUE^®^ High Density Barium Sulfate 98.75%, Bracco imaging, Milan, Italy), and porcine skin gelatin (Gelatin 48723, Sigma-Aldrich, Darmstadt, Germany), previously filtered and warmed to 60 °C. Perfusion was stopped when the contrast solution was clearly seen in the capillary bed of the placenta. Thereafter, placentas were separated from the conceptuses and immersed in 4% PFA for 24 h, washed, and stored in PBS. A total of 16 placentas (eight FGR and eight controls) from four different litters were included for analysis.

X-ray computed microtomography scans were performed using SkyScan 1172 micro-CT system (Bruker microCT, Kontich, Belgium) with X-ray energy of 80 keV. The image pixel size was set to 8.98 µm and a 0.5 mm Al filter was applied. Projection images were captured between the rotation steps of 0.2°. Each projection was merged from 6 or 8 images averaged from 5 frames to minimize the image noise and captured with different field of view to cover the entire sample, resulting in 3872 by 3872–4956 pixels. The total scan duration was up to 15 h.

For image reconstruction, GPU powered NRecon software (v.1.7.3.2, Bruker micro-CT) was used. Image processing and analysis was done by CT analyser software (v.1.20.3.0, Bruker micro-CT,). The reconstructed image stacks were median filtered with 2 pixels circular kernel, and histogram based manual global threshold was applied. The distribution of vessels thickness, and parameters of volume and surface of vascular tree were calculated. Additionally, color-coding visualization of the local thickness was applied. An observer blinded to group assignment (B.L.) performed all evaluations.

### Neurobehavioral assessment

On postnatal day 1 (PND1), kittens underwent a validated NBA protocol^18,73^. Short-term motor assessment comprised scoring of gait, posture, locomotion, head and limb activity, and activity duration. Afterwards, the cranial nerves, pain response, and righting reflex were tested for sensory evaluation. All assessments were filmed and later scored by an observer blinded to the groups (I.V.). A full description of NBA protocols can be found in the Supplementary materials.

### Brain harvesting

Immediately after NBA, animals were deeply sedated with IM ketamine (35 mg/kg) and xylazine (6 mg/kg), and transcardially perfused with 0.9% saline + heparin (100 u/mL; 3 min at 30 mL/min) followed by 4% PFA (5 min at 30 ml/min). Their brains were removed from the skull and further immerse fixed in 4% PFA for 48 h. A subgroup of kittens (n = 23) was perfused with heparinized saline, followed by 4% PFA and 2% (10 mM) gadoteridol (ProHance^®^ 1 mL of 279.3 mg/mL solution). Their brains were kept inside their craniums for a maximum of 7 days to undergo MRI, after which they were removed and processed for histological assessment.

### Brain MRI

Ex vivo MRI was performed on perfused fixed brains using the active staining technique. Briefly, a Bruker Biospec 9.4 Tesla small animal MR scanner (Bruker Biospin, Ettlingen, Germany; horizontal bore, 20 cm) equipped with actively shielded gradients (600 mT/m) was used. Data was acquired using a 72 mm internal diameter quadrature volume coil for transmission decoupled with a rat brain quadrature shaped surface coil for signal reception (volume resonator, Rapid Biomedical, Rimpar, Germany). Data was acquired with a high-resolution 3D Flash sequence (TE/TR 5.5/50 ms; flip angle 70 degrees; slice thickness 0.35 mm with no interslice gap, data matrix 392 × 392 × 392; isotropic resolution 89 μm; 4 averages, acquisition time 1h15min) and a SE-EPI sequence (8 segments; TE/TR: 25/150 ms; slice thickness 0.4 mm with no interslice gap, data matrix 192 × 160 × 160; isotropic spatial resolution of 208 μm; 64 directions and 3 b-values per direction of 800, 1000, 1500 s/mm^2^, acquisition time 11 h 43 min).

MRI volumes were processed to quantify microstructural properties in the following brain regions: frontal cortex, corpus callosum, caudate nucleus, putamen, internal capsule, corona radiata, hippocampus, thalamus, and hypothalamus. Automatic atlas-based parcellation was obtained by diffeomorphic registration of the atlas template in Ferraris et al. ^74^ to the acquired structural images, and then translated to the diffusion space. Fractional anisotropy (FA) and mean diffusivity (MD) were estimated using dipy python libraries^75,76^, and their mean values in regions of interest were computed. An observer blinded to group assignment (E.M.) performed all evaluations. A complete description of MRI processing can be found in Supplementary materials.

Of 23 acquisitions (13 controls 10 FGR), 4 (2 in each group) were excluded from analysis due to important artifacts. Statistical comparison between groups was performed in one random cerebral hemisphere. An interim power calculation, based on the FA data in the hippocampus, confirmed a power of 95% to detect these differences.

### Brain histology

Following fixation, brains were paraffin embedded and serially sectioned at 4 µm. Three sets of four serial coronal sections every 100 µm were taken at each of the following two levels, as previously described^18^: level 1 started at the medial septal nucleus and level 2 at the hippocampal formation.

Six slides per brain (three slides per level) were stained with Cresyl Violet (CV; C5042-10G; Sigma-Aldrich, Overijse, Belgium), and two slides per brain (one slide per level, eight slides in total) were incubated with each of the following four primary antibodies: mouse monoclonal anti-human Ki67 (M724001-2; Agilent, Diegem, Belgium), mouse monoclonal anti-glial fibrillary acidic protein antibody (GFAP) (G6171, Sigma-Aldrich, St Louis, MO, USA), anti-NG2 chondroitin sulfate proteoglycan antibody (MAB5384, Millipore, Billerica, MA, USA), or a terminal deoxynucleotidyl transferase dUTP nick end labeling (TUNEL) method for fluorescent in situ end labeling of double- stranded DNA fragmentation (Apoptag S7110; Millipore). The secondary antibody was Alexa Fluor^®^ 488 goat anti-mouse conjugate (Invitrogen) or Alexa Fluor^®^ 647 goat anti-mouse conjugate. Sections were counterstained with Hoechst 33342 (Sigma-Aldrich, Bornem, Belgium). Eight brain areas were assessed, namely, the frontal cortex, corpus callosum, caudate nucleus, putamen, hippocampus (CA1, CA3, dentate gyrus), and thalamus (anteroventral nucleus).

Details on image acquisition and quantification can be found in Supplementary materials.

### Pulmonary function testing

Pressure–volume and forced oscillation maneuvers were performed via a tracheostomy on PND1 using the FlexiVent system (SciReq; FlexiVent, Montreal, QC, Canada). Animals were sedated with ketamine (35 mg/kg) and xylazine (6 mg/kg) before tracheostomy. An 18-gauge metal cannula was inserted into the trachea and secured with an airtight suture. Kittens were ventilated with a tidal volume of 10 mL/kg and positive end-expiratory pressure of 3 cmH2O at a rate of 120 breaths/min. Before lung function tests, two deep inflation maneuvers were performed until reaching a pressure of 30 cmH2O to maximally inflate the lungs and standardize lung volume. Both pressure–volume (inspiratory capacity, static compliance, and static elastance) and forced oscillation tests (tissue damping, tissue elastance, central airway resistance, respiratory system resistance, dynamic compliance, and dynamic elastance) were performed as previously described^77^. The mean of three separate measurements for each maneuver, with a coefficient of determination > 95%, was calculated and used as a single data point for analysis.

### Histological lung assessment

Lungs were processed and assessed in accordance with international guidelines^78^. Briefly, after PFT, lungs were removed en bloc via thoracotomy, the trachea was cannulated with a 20-gauge catheter, and the left lung was pressure fixed for 24 h at a constant hydrostatic pressure of 25 cmH2O in 4% PFA^77^. After fixation, left lung volume was calculated using water displacement before paraffin embedding. Alveolar morphology was measured on digitally scanned 5 µm hematoxylin and eosin (H&E)-stained slides using a semi-automated, validated Fiji-plugin (ImageJ) (http://fiji.sc/Fiji) that randomly selected 20 fields per lung^79^, according to stereological principles. Calculations of mean linear intercept (Lm), alveolar air space (Lma), and interalveolar septal thickness (Lmw) were made as previously described^29^. Vascular morphology was evaluated using immunohistochemistry. A primary α-smooth muscle actin (α-SMA) antibody (mouse anti-human, M0851; DakoCytomation, Glostrup, Denmark) was used in combination with a horseradish peroxidase-conjugated secondary antibody (goat anti-mouse, 115-035-044; Jackson ImmunoResearch, Ely, UK). Aminoethyl carbazole was used as a chromogen. A minimum of 10 pulmonary arteries with an external diameter of 30–100 μm per lung slide were examined, measuring both internal and external diameter of the media to calculate the vascular medial thickness^27^. An observer blinded to group assignment (D.B.) performed all pulmonary histological evaluations.

### Statistical analysis

Data were analysed and graphed using RStudio (RStudio: Integrated Development for R. RStudio, PBC, Boston, MA, USA) and Prism 9 for MacOS (GraphPad Prism, San Diego, CA, USA). Data distribution was checked for normality and presented as mean with standard deviation or median with interquartile range, as appropriate. Survival data was assessed by Fisher’s exact; all other parameters were analysed using a linear mixed-effects model, considering the mother as a random effect and the presence of FGR as a fixed effect. A p value of < 0.05 was considered significant.

## Supplementary Information

Supplementary Information.

## Data Availability

The datasets analysed during the current study available from the corresponding author on reasonable request.
