# Supplementary material for: Placental vascular alterations are associated with early neurodevelopmental and pulmonary impairment in the rabbit fetal growth restriction model
Source: Sci Rep. 2022 Nov 16;12:19720. doi: 10.1038/s41598-022-22895-6 (PMC9668827; doi:10.1038/s41598-022-22895-6)
Supplement: Supplementary file 1 — Supplementary Information. [file 41598_2022_22895_MOESM1_ESM.pdf]

Placental vascular alterations are associated with early neurodevelopmental and pulmonary impairment in the rabbit fetal growth restriction model

Ignacio Valenzuela, David Basurto, Yannick Regin, Andre Gie, Lennart van der Veeken, Simen Vergote, Emma Muñoz-Moreno, Bartosz Leszczynski, Birger Tielemans, Greetje Vande Velde, Jan Deprest, Johannes van der Merwe.

Supplementary information

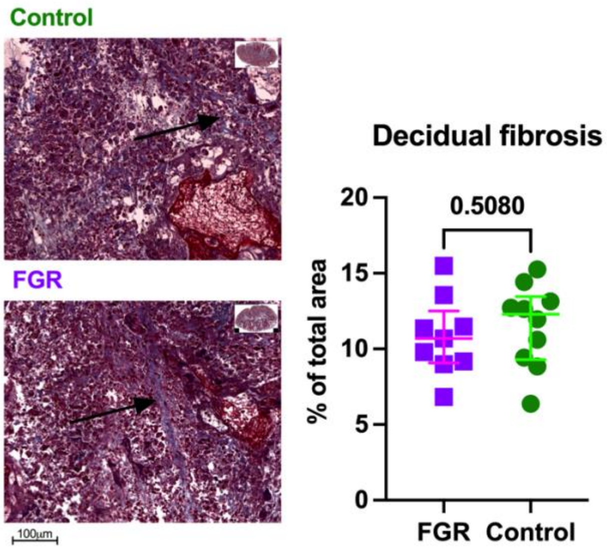

Supplementary Figure S1. Area of decidua with collagen depositions, in Masson-Trichrome stained placentas at gestational day 30. Data from 20 placentas (10 litters, 1 control and 1 FGR per litter), analyzed by linear mixed model, bars show median and IQR.

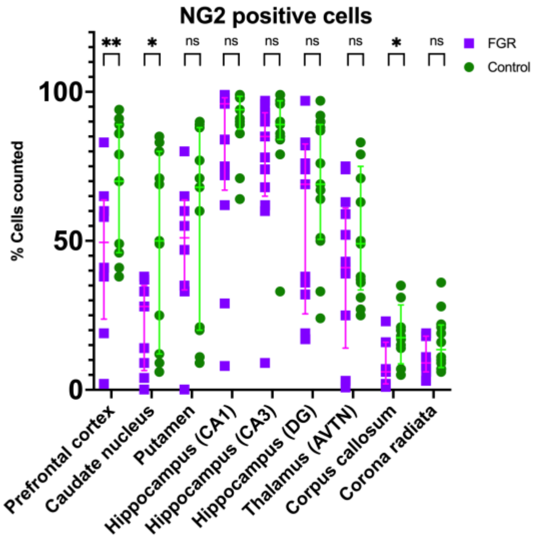

Supplementary Figure S2. Expression of NG2 in different brain regions, in 14 FGR and 19 control postnatal day 1 brains from 11 litters. CA1: cornu ammonis 1; CA3: cornu ammonis 3; DG: dentate gyrus; AVTN; anteroventral thalamic nuclei. Data were analysed using a linear mixed effects model, bars show median and IQR.

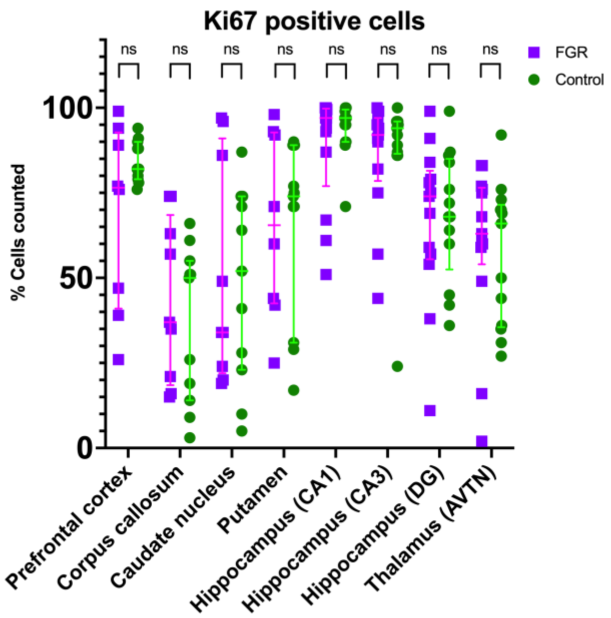

Supplementary Figure S3. Expression of Ki-67 in different brain regions, in 14 FGR and 19 control postnatal day 1 brains from 11 litters. CA1: cornu ammonis 1; CA3: cornu ammonis 3; DG: dentate gyrus; AVTN; anteroventral thalamic nuclei. Data were analysed using a linear mixed effects model, bars show median and IQR.

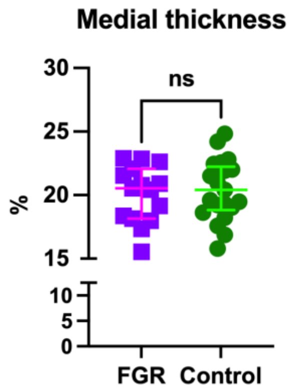

Supplementary Figure S4. Medial thickness in peripheral pulmonary arteries, expressed as the percentage of total vessel diameter, in 15 FGR and 19 control postnatal day 1 lungs from 6 litters. Data were analysed using a linear mixed effects model, bars show median and IQR.

Table S1. Umbilical artery doppler parameters at gestational day 30.

Parameter	FGR (n=10)		Control (n=18)		p-value
	Mean	SE	Mean	SE	
Peak systolic velocity, cm/s	175.05	16.33	182.26	17.10	0.663
End diastolic velocity, cm/s	21.23	8.34	26.14	2.07	0.561
Velocity time integral	18.91	2.56	20.81	2.30	0.467
Mean velocity, cm/s	86.21	7.27	91.02	8.42	0.516
Pulsatility index	1.85	0.14	1.77	0.09	0.577
Resistance index	0.89	0.03	0.87	0.02	0.666

Ultrasound was performed in fetuses at the ovarian ends of both uterine horns, from 10 litters. Data were analysed by linear mixed-effects model.

Table S2. Placental histological assessment

Parameter	FGR	Control	p-value
**Placental zones, %**			
Labyrinth	38.9 (34-45)	45.9 (42-51)	<0.0001
Junction	16.6 (13-19)	16.3 (13-18)	0.7240
Decidua	44.7 (39-51)	37.5 (33-43)	<0.0001
**Placental zones, volume (mm^3)**			
Labyrinth	2.028 ± 0.562	2.843 ± 0.722	<0.0001
Junction	0.862 ± 0.292	1.024 ± 0.328	0.0032
Decidua	2.231 ± 0.517	2.267 ± 0.642	0.7350
**Labyrinth cells, %**			
Fetal capillaries	27.2 (23-32)	44.3 (39-48)	<0.0001
Trophoblast	32.8 (26-39)	29.6 (25-35)	0.1090
Maternal blood spaces	39.7 (36-43)	26.1 (22-28)	<0.0001
**Labyrinth cells, volume (mm^3)**			
Fetal capillaries	0.566 ± 0.188	1.442 ± 0.431	<0.0001
Trophoblast	0.663 ± 0.16	0.933 ± 0.205	0.0042
Maternal blood spaces	0.824 ± 0.258	0.867 ± 0.351	0.7542

Placentas were obtained at caesarian section at gestational day 30. For placental zone assessment, 51 FGR and 59 control placentas from 20 litters were used. For labyrinth structures assessment, 10 FGR and 10 control placentas from 10 litters were used. Data were analysed using a linear mixed-effects model, displayed as median (IQR) or mean ± SD.

Table S3. Neuropathological assessment in postnatal day 1 brains.

Parameter	FGR	Control	p-value
**Neuron density, cells/μm^2**			
Frontal cortex	0.0059 ± 0.002	0.0088 ± 0.003	<0.0001
Corpus callosum	0.0021 ± 0.001	0.0031 ± 0.001	0.0059
Caudate nucleus	0.0100 ± 0.003	0.0127 ± 0.005	<0.0001
Putamen	0.0064 ± 0.002	0.0085 ± 0.002	0.0018
Hippocampus CA1	0.0111 ± 0.003	0.0158 ± 0.003	<0.0001
Hippocampus CA3	0.0097 ± 0.002	0.0147 ± 0.002	<0.0001
Hippocampus DG	0.0092 ± 0.003	0.0127 ± 0.003	<0.0001
AVTN	0.0066 ± 0.003	0.0098 ± 0.003	0.0170
**TUNEL, % of positive cells**			
Corpus callosum	4.46 (2.7- 5.9)	2.58 (1.2-3.4)	0.0127
Caudate nucleus	0.85 (0.5-1.5)	0.80 (0.6-0.9)	0.1560
Hippocampus CA1	1.2 (0.4-1.9)	0.2 (0-0.5)	0.0005
Hippocampus CA3	1.99 (0.8-2.7)	0.75 (0-1.2)	0.0004
Hippocampus DG	0.44 (0.2-0.5)	0.40 (0.1-0.5)	0.4828
AVTN	0.81 (0.2-1.1)	0.31 (0-0.5)	0.0020
Hippocampus	3.74 (2.7-4.9)	1.78 (0.3-3.1)	0.0003
**GFAP, % of positive cells**			
Corpus callosum	31.9 (21.5-38.0)	21.5 (15.6-24.4)	<0.0001
Caudate nucleus	0.63 (0.2-1.1)	0.37 (0.1-0.5)	0.0305
Hippocampus CA1	5.4 (1.7-6.8)	3.3 (0.9-5.4)	0.1549
Hippocampus CA3	15 (8.9-20)	11.7 (6.8-16.7)	0.2190
Hippocampus DG	15.6 (11.5-20.7)	11.5 (5.7-12.6)	0.0168
AVTN	4.0 (0.4-2.8)	2.2 (0.3-1.6)	0.1210
Hippocampus	11.4 (7.9-14.1)	10.8 (6.4-12.4)	0.6850
**NG2, % of positive cells**			
Frontal cortex	49.5 (24-64)	70 (46-89)	0.0093
Corpus callosum	6 (2-16)	17.5 (9-29)	0.0423
Caudate nucleus	28 (7-35)	50 (12-80)	0.0245
Putamen	51 (34-64)	68 (20-88)	0.5630
Hippocampus CA1	96 (67-98)	94 (88-99)	0.2440
Hippocampus CA3	85 (65-93)	89 (84-97)	0.3580
Hippocampus DG	69 (26-83)	69 (51-89)	0.5020
AVTN	41 (14-61)	49 (34-75)	0.4181
Corona radiata	9 (6-18)	13.5 (8-22)	0.2588
**Ki67, % of positive cells**			
Frontal cortex	76.5 (41-93)	82 (79-90)	0.0759
Corpus callosum	37 (19-69)	50 (14-55)	0.3713
Caudate nucleus	34 (22-91)	52 (23-74)	0.9015
Putamen	65.5 (43-93)	74 (31-89)	0.883
Hippocampus CA1	97 (77-100)	97 (90-100)	0.221
Hippocampus CA3	92 (79-97)	94 (87-96)	0.662
Hippocampus DG	74 (56-82)	68 (53-85)	0.891
AVTN	63 (54-77)	66 (36-72)	0.809

Neuron density from 27 FGR and 29 control subjects from 16 litters. Expression of TUNEL, GFAP, NG2 and Ki67 was measured in 14 FGR and 19 control brains from 11 litters. Data were analysed using a linear mixed-effects model, displayed as mean ± SD for neuron density, and median (IQR) for all other parameters. AVTN, anteroventral thalamic nucleus; CA1, cornu ammoni 1; CA3, cornu ammoni 3; DG, dentate gyrus.

Table S4. Diffusion tension metrics in ex vivo brain magnetic resonance imaging

Parameter	FGR	Control	p-value
**Fractional anisotropy**			
Frontal cortex	0.402 (0.356-0.446)	0.4543(0.412-0.466)	0.0374
Hippocampus	0.390 (0.367-0.414)	0.456 (0.394-0.469)	0.0259
Caudate nucleus	0.360 (0.331-0.383)	0.429 (0.334-0.515)	0.111
Putamen	0.316 (0.252-0.381)	0.382 (0.291-0.410)	0.108
Thalamus	0.294 (0.262-0.318)	0.369 (0.278-0.374)	0.0326
Hypothalamus	0.365 (0.287-0.444)	0.432 (0.347-0.517)	0.170
Corpus callosum	0.411 (0.379-0.456)	0.497 (0.461-0.560)	0.0032
Internal capsule	0.367 (0.295-0.427)	0.457 (0.421-0.523)	0.0202
Corona radiata	0.395 (0.347-0.442)	0.507 (0.459-0.563)	0.0003
Whole brain	0.373 (0.357-0.391)	0.421 (0.389-0.430)	0.0444
**Mean Diffusivity**			
Frontal cortex	0.0004 (0.00038-0.0006)	0.0005 (0.0004-0.00057)	0.394
Hippocampus	0.0003 (0.0003-0.0003)	0.0004 (0.0003-0.00036)	0.004
Caudate nucleus	0.0003 (0.00027-0.0003)	0.0003 (0.0003-0.00037)	0.128
Putamen	0.0003 (0.00026-0.0003)	0.0003 (0.0003-0.00034)	0.572
Thalamus	0.0003 (0.00027-0.0003)	0.0003 (0.0003-0.00033)	0.352
Hypothalamus	0.0003 (0.00023-0.0004)	0.0003 (0.0003-0.00034)	0.582
Corpus callosum	0.0003 (0.00027-0.0003)	0.0003 (0.0003-0.00034)	0.197
Internal capsule	0.0003 (0.00022-0.0003)	0.0003 (0.0002-0.00031)	0.286
Corona radiata	0.0003 (0.00026-0.0003)	0.0003 (0.0003-0.00034)	0.151

Factional anisotropy and mean diffusivity were measured in 11 FGR and 8 control brains from 6 litters, at postnatal day 1. Data were analysed using a linear mixed-effects model, displayed as median and (IQR).

Table S5. Pulmonary function tests and lung histology results.

Parameter	FGR (n=19)	Control (n=21)	p-value
**Functional tests**			
Inspiratory capacity/body weight, mL/kg	31.60 ± 5.802	34.13 ± 4.552	0.0823
Static compliance/body weight, mL/(cmH_20•kg)	2.127 ± 0.4762	2.618 ± 0.5008	0.0018
Hysteresis (A), mL•cmH_2O	1.450 ± 0.5405	2.161 ± 0.6564	0.0004
Tissue elastance (H), cmH_2O/mL	10.96 ± 3.021	7.020 ± 1.870	<0.0001
Tissue damping (G), cmH_2O/mL	2.961 ± 0.7466	1.948 ± 0.4589	<0.0001
Respiratory system resistance, cmH_2O•s/mL	0.3863 ± 0.09244	0.2590 ± 0.06326	<0.0001
Central airway resistance, cmH_2O•s/mL	0.0503 ± 0.0458	0.0666 ± 0.02130	0.5350
Dynamic compliance, mL/cmH_2O	0.059 ± 0.007	0.103 ± 0.006	<0.0001
**Morphometry**			
Alveolar size (Lm), µm	79.77 ± 6.70	74.31 ± 5.53	0.0049
Airspace size (Lma), µm	63.43 ± 6.730	58.49 ± 6.707	0.0191
Septal thickness (Lmw), µm	16.49 (14.1-16.4)	15.80 (13.7-17.6)	0.3430
Alveolar surface area, cm^2	569.4 ± 221.2	938.7 ± 282.1	0.0004
Medial thickness, %	20.53 (18-1-22.1)	20.41 (18.8-22.2)	0.3300

Pulmonary assessment in postnatal day 1. Pulmonary function tests and morphometry results from 19 FGR and 21 control subjects from 6 litters. Data were analysed using a linear mixed-effects model, displayed as mean ± SD or median (IQR).

MATERIALS AND METHODS

Placental immunohistochemistry

Sections were dewaxed and rehydrated to tris-buffered saline (TBS, which was used for all wash steps further in the protocol). Antigen retrieval was performed using Pepsin (Sigma, 0.04% in preheated 0.01 M HCl, 10min, 37 °C). Slides were washed in TBS (once at 4 °C and then twice at RT). Non-specific binding was blocked using blocking buffer (dH_2O containing 2% bovine serum albumin, 1% skimmed dry milk and 0.1% Tween20, 15min, 3.3% Normal Goat Serum, RT). Sections were then incubated with Rabbit anti-Cytokeratin MNF116 (Agilent M0821;1:200 (0.8µg/ml); 2h 37°C). Bound antibody was detected with Goat anti-Mouse (Abcam; ab102445; 1:25; 30 min.) attached with APAAP Complex (STAR67; Bio-Rad; 1:50), which converts Fast Blue BB (F3378; Sigma) into a blue product. Sections were washed in TBS and then endogenous peroxidase activity was blocked with hydrogen peroxide (3% in methanol) for 30min at room temperature (RT). Sections were washed and then incubated with blocking buffer containing 10% goat serum for 15 min at RT. Slides were incubated with biotinylated lectin (isolectin B4, B-1205, Vector Laboratories; 1:100, 90min, 37 °C) before detection with horseradish peroxide-conjugated streptavidin (P0397, Agilent; 1:840, 30 min, RT) followed by 3′-*Diaminobenzidine* (Sigma, 20 min RT). Slides were washed in water and counterstained with Nuclear Fast Red (Vector laboratories, 5min) before dehydrating and mounting with Neo-Clear.

Neurobehavioral assessment

Neurobehavioral PND1 evaluation based on a modification of neurobehavioral scoring protocol previously described[1]. For each animal, testing is videotaped and scored by a blinded observer. The kittens are evaluated in a designated space close to their pen with auditory and olfactory contamination kept to a minimum. Before handling they remain undisturbed in this assessment area for a 3-5min adaptation period.

- Cranial nerves are assessed by testing smell (olfaction is tested by recording time to aversive response to a cotton swab soaked with pure ethanol), sucking, and swallowing (by introduction of formula into the kittens' mouth with a plastic syringe), and head turn to feeding. The responses are graded on a scale of 0 to 3, 0 being the worst response and 3 the best response.
- Motor examination includes tone, motor activity, and locomotion on a flat surface, righting reflex, and gait. The righting reflex is assessed when the kittens are placed on their backs and the number of times turned prone (within 2s) from supine position in 5 tries is registered. Gait is examined based on a modification by Georgiadis et al.[2] Locomotion is assessed as described by Kannan et al.[3]
- Sensory examination is limited to touch on the face (touching the face with cotton swab on both sides) and extremities as well as pain on limbs (mild pin prick).

MRI processing

MRI volumes were processed to quantify microstructural properties in specific brain regions. First, automatic atlas-based parcellation was performed on the structural T1-weighted images of each subject. To skull-strip the images, and remove non-brain tissues a brain mask was manually delineated in one of the subjects randomly chosen as reference. The brain mask was adapted to the other subjects' structural images by means of the diffeomorphic registration algorihm implemented in ANTs[4]. The resulting brain mask for each subject were applied to skull-strip the images. Afterwards, the atlas

template of the neonatal rabbit brain atlas in Ferraris et al.[5] was registered to the structural image of each subject using agein the diffeomorphic registration algorihm in ANTs. The obtained transformation was applied to the label maps obtained from the atlas to identify regions of interest in each subjects' brain.

The diffusion weigthed MRI was pre-processed using dipy[6] including denoising and eddy current corrections based on elastic registration to the anatomical image. A diffusion tensor model was fitted to the data and fractional anisotropy (FA) and mean diffusivity (MD) maps were obtained. Also NODDI (neurite orientation dispersion and density imaging) was obtained from the diffusion weighted imaging[7], and neurite density (ND), orientation dispersion index (ODI) and isotropic volume fraction (ISO) were estimated using dmipy[8].

For each subject, the brain parcellation obtained for the structural T1-weighted images was translated to the diffusion space, and regional FA, MD, ND, ODI and ISO were computed as the average value in each region of interest.

Neuropathology: Image acquisition and quantification

Histological slides were digitized using the Zeiss AxioScan Z1 imaging platform (AxioScan Slide Scanner, Carl Zeiss MicroImaging GmbH, Munich, Germany), using a 20x Plan Apochromat objective coupled to a 3 Chip CCD Camera (Hamamatsu Photonics, Japan). All focusing and field-of-view assembly was done by the integrated Carl Zeiss Zen software (Carl Zeiss MicroImaging GmbH, Munich, Germany).

Quantification of neuron density and immunohistochemistry-positive cells was done according to previous reports by our group[9]. Briefly, for neuronal quantification, regions of interest (ROI) were selected on 3 consecutive slides per level separated by 100 µm. In each ROI, 5 squares (100 x 100-µm) were selected at low magnification so individual cells were not visible to avoid bias. Neurons were manually counted in these squares, and neuron density was calculated dividing the total number of cells by the area.

For quantification of the immunohistochemistry-positive cells in the TUNEL, GFAP, NG2 and Ki-67 stains quantification profiles on the digitized whole-slide images were obtained with QuPath software[10]. Herein the whole ROI was delineated, and quantification was done by using the fast cell counting and positive cell detection functions. Positive cell counts were expressed as counts per area and/or percentage of positive cells per total cells detected.

REFERENCES

1 Derrick, M. *et al.* Preterm fetal hypoxia-ischemia causes hypertonia and motor deficits in the neonatal rabbit: a model for human cerebral palsy? *J Neurosci* **24**, 24-34, doi:10.1523/JNEUROSCI.2816-03.2004 (2004).

2 Georgiadis, P. *et al.* Characterization of acute brain injuries and neurobehavioral profiles in a rabbit model of germinal matrix hemorrhage. *Stroke* **39**, 3378-3388, doi:10.1161/STROKEAHA.107.510883 (2008).

3 Kannan, S. *et al.* Dendrimer-based postnatal therapy for neuroinflammation and cerebral palsy in a rabbit model. *Sci Transl Med* **4**, 130ra146, doi:10.1126/scitranslmed.3003162 (2012).

4 Avants, B. B., Epstein, C. L., Grossman, M. & Gee, J. C. Symmetric diffeomorphic image registration with cross-correlation: evaluating automated labeling of elderly and neurodegenerative brain. *Med Image Anal* **12**, 26-41, doi:10.1016/j.media.2007.06.004 (2008).

5 Ferraris, S. *et al.* A magnetic resonance multi-atlas for the neonatal rabbit brain. *Neuroimage* **179**, 187-198, doi:10.1016/j.neuroimage.2018.06.029 (2018).

6 Garyfallidis, E. *et al.* Dipy, a library for the analysis of diffusion MRI data. *Front Neuroinform* **8**, 8, doi:10.3389/fninf.2014.00008 (2014).

7 Zhang, H., Schneider, T., Wheeler-Kingshott, C. A. & Alexander, D. C. NODDI: practical in vivo neurite orientation dispersion and density imaging of the human brain. *Neuroimage* **61**, 1000-1016, doi:10.1016/j.neuroimage.2012.03.072 (2012).

8 Fick, R. H. J., Wassermann, D. & Deriche, R. The Dmipy Toolbox: Diffusion MRI Multi-Compartment Modeling and Microstructure Recovery Made Easy. *Front Neuroinform* **13**, 64, doi:10.3389/fninf.2019.00064 (2019).

9 Van der Veeken, L. *et al.* Maternal surgery during pregnancy has a transient adverse effect on the developing fetal rabbit brain. *American journal of obstetrics and gynecology* **221**, 355 e351-355 e319, doi:https://doi.org/10.1016/j.ajog.2019.07.029 (2019).

10 Bankhead, P. *et al.* QuPath: Open source software for digital pathology image analysis. *Scientific reports* **7**, 16878, doi:10.1038/s41598-017-17204-5 (2017).
